# Author Correction: Inferring time-varying generation time, serial interval, and incubation period distributions for COVID-19

**DOI:** 10.1038/s41467-023-35855-z

**Published:** 2023-01-10

**Authors:** Dongxuan Chen, Yiu-Chung Lau, Xiao-Ke Xu, Lin Wang, Zhanwei Du, Tim K. Tsang, Peng Wu, Eric H. Y. Lau, Jacco Wallinga, Benjamin J. Cowling, Sheikh Taslim Ali

**Affiliations:** 1 WHO Collaborating Centre for Infectious Disease Epidemiology and Control, School of Public Health, Li Ka Shing Faculty of Medicine, The University of Hong Kong, Hong Kong Special Administrative Region, China; 2 Laboratory of Data Discovery for Health Limited, Hong Kong Science and Technology Park, New Territories, Hong Kong Special Administrative Region, China; 3 College of Information and Communication Engineering, Dalian Minzu University, Dalian, 116600 China; 4 Department of Genetics, University of Cambridge, Cambridge, CB2 3EH UK; 5 Center for Infectious Disease Control, National Institute for Public Health and the Environment (RIVM), Bilthoven, The Netherlands; 6 Department of Biomedical Data Sciences, Leiden University Medical Center, Leiden, The Netherlands

**Keywords:** Infectious diseases, Epidemiology, Statistical methods, SARS-CoV-2

Correction to: *Nature Communications* 10.1038/s41467-022-35496-8, published online 13 December 2022

The original version of this Article contained an error in the authorship tagging statements for Dongxuan Chen, Yiu-Chung Lau, and Xiao-Ke Xu, which were previously given as ‘These authors jointly supervised this work’. The correct version states ‘These authors contributed equally’. This has been updated in both the PDF and HTML versions of the Article.

