# Supplementary material for: Inferring time-varying generation time, serial interval, and incubation period distributions for COVID-19
Source: Nat Commun. 2022 Dec 13;13:7727. doi: 10.1038/s41467-022-35496-8 (PMC9747081; doi:10.1038/s41467-022-35496-8)
Supplement: Supplementary file 2 — Reporting Summary [file 41467_2022_35496_MOESM2_ESM.pdf]

nature portfolio

Corresponding author(s): NCOMMS-22-31916C Prof. Benjamin J Cowling

Last updated by author(s): Nov 28, 2022

Reporting Summary

Nature Portfolio wishes to improve the reproducibility of the work that we publish. This form provides structure for consistency and transparency in reporting. For further information on Nature Portfolio policies, see our Editorial Policies and the Editorial Policy Checklist.

Statistics

For all statistical analyses, confirm that the following items are present in the figure legend, table legend, main text, or Methods section.

n/a	Confirmed	
☐	☒	The exact sample size (n) for each experimental group/condition, given as a discrete number and unit of measurement
☐	☒	A statement on whether measurements were taken from distinct samples or whether the same sample was measured repeatedly
☐	☒	The statistical test(s) used AND whether they are one- or two-sided<br>*Only common tests should be described solely by name; describe more complex techniques in the Methods section.*
☐	☒	A description of all covariates tested
☐	☒	A description of any assumptions or corrections, such as tests of normality and adjustment for multiple comparisons
☐	☒	A full description of the statistical parameters including central tendency (e.g. means) or other basic estimates (e.g. regression coefficient) AND variation (e.g. standard deviation) or associated estimates of uncertainty (e.g. confidence intervals)
☒	☐	For null hypothesis testing, the test statistic (e.g. F, t, r) with confidence intervals, effect sizes, degrees of freedom and P value noted<br>*Give P values as exact values whenever suitable.*
☒	☐	For Bayesian analysis, information on the choice of priors and Markov chain Monte Carlo settings
☒	☐	For hierarchical and complex designs, identification of the appropriate level for tests and full reporting of outcomes
☒	☐	Estimates of effect sizes (e.g. Cohen's d, Pearson's r), indicating how they were calculated

Our web collection on statistics for biologists contains articles on many of the points above.

Software and code

Policy information about availability of computer code

Data collection	No software was used for this study's data collection. As we used the secondary data based on previous studies that were published by Ali et al: Science. 2020;369(6507):1106-1109. doi:10.1126/science.abc9004 and Liu et al: Scientific Data. 2021;8(1). doi:10.1038/s41597-021-00844-8
Data analysis	All statistical analyses were conducted in R version 4.0.4 (R Development Core Team, 2011). R codes and list of the packages used are provided in the Github repository (https://github.com/DxChen0126/time_varying_GT)<br>R packages for data analysis and visualization of this work include:<br>doParallel (version 1.0.16); foreach (version 1.5.1); fitdistrplus (version 1.1-6); mixdist (version 0.5-5); incidence (version 1.7.3); EpiEstim (version 2.2-4); ggplot2 (version 3.3.5); ggbreak (version 0.0.9); ggpubr (version 0.4.0); cowplot (version 1.1.1); latex2exp (version 0.9.0)

For manuscripts utilizing custom algorithms or software that are central to the research but not yet described in published literature, software must be made available to editors and reviewers. We strongly encourage code deposition in a community repository (e.g. GitHub). See the Nature Portfolio guidelines for submitting code & software for further information.

Data

Policy information about availability of data

All manuscripts must include a data availability statement. This statement should provide the following information, where applicable:

- Accession codes, unique identifiers, or web links for publicly available datasets
- A description of any restrictions on data availability
- For clinical datasets or third party data, please ensure that the statement adheres to our policy

We used the line list data as reported by China's municipal health commissions outside Hubei province from January 1 to February 29, 2020. The original data was extracted from the publicly available case reports provided by more than 200 municipal health commissions in Mainland China.
All the data used in the analysis are available at Github: https://github.com/DxChen0126/time_varying_GT

Human research participants

Policy information about studies involving human research participants and Sex and Gender in Research.

Reporting on sex and gender	Not applicable
Population characteristics	Not applicable
Recruitment	Not applicable
Ethics oversight	Not applicable

Note that full information on the approval of the study protocol must also be provided in the manuscript.

Field-specific reporting

Please select the one below that is the best fit for your research. If you are not sure, read the appropriate sections before making your selection.

☒ Life sciences ☐ Behavioural & social sciences ☐ Ecological, evolutionary & environmental sciences

For a reference copy of the document with all sections, see nature.com/documents/nr-reporting-summary-flat.pdf

Life sciences study design

All studies must disclose on these points even when the disclosure is negative.

Sample size	The study was based on the population based epidemiological parameters, which were evaluated by using daily confirmed COVID-19 cases and their line-list information. The temporal estimates of these parameters were performed under sufficient sample size (i.e. we set at least 20 infectors with complete exposure information) in a given time window. Therefore no sample size calculation is required for this study.
Data exclusions	We excluded the cases in with no confirmed transmission pairs for main analysis.
Replication	We provided the data and related codes for the replication and available at https://github.com/DxChen0126/time_varying_GT
Randomization	This is an observational study and no randomization is required.
Blinding	This is an observational study and no blinding is required.

Reporting for specific materials, systems and methods

We require information from authors about some types of materials, experimental systems and methods used in many studies. Here, indicate whether each material, system or method listed is relevant to your study. If you are not sure if a list item applies to your research, read the appropriate section before selecting a response.

Materials & experimental systems

n/a	Involved in the study
☒	☐ Antibodies
☒	☐ Eukaryotic cell lines
☒	☐ Palaeontology and archaeology
☒	☐ Animals and other organisms
☒	☐ Clinical data
☒	☐ Dual use research of concern

Methods

n/a	Involved in the study
☒	☐ ChIP-seq
☒	☐ Flow cytometry
☒	☐ MRI-based neuroimaging
